# Support for the Microgenderome: Associations in a Human Clinical Population

**DOI:** 10.1038/srep19171

**Published:** 2016-01-13

**Authors:** Amy Wallis, Henry Butt, Michelle Ball, Donald P. Lewis, Dorothy Bruck

**Affiliations:** 1 Psychology Department, Victoria University, Victoria, Australia; 2 Bioscreen (Aust) Pty Ltd, Victoria, Australia; 3 CFS Discovery Clinic, Donvale, Victoria, Australia

## Abstract

The ‘microgenderome’ provides a paradigm shift that highlights the role of sex differences in the host-microbiota interaction relevant for autoimmune and neuro-immune conditions. Analysis of cross-sectional self-report and faecal microbial data from 274 patients with Myalgic Encephalomyelitis/Chronic Fatigue Syndrome (ME/CFS) suggests that commensal gut microorganisms may play both protective and deleterious roles in symptom expression. Results revealed significant sex-specific interactions between *Firmicutes* (*Clostridium, Streptococcus, Lactobacillus and Enterococcus*) and ME/CFS symptoms (including neurological, immune and mood symptoms), regardless of compositional similarity in microbial levels across the sexes. Extending animal studies, we provide support for the microgenderome in a human clinical population. Applied and mechanistic research needs to consider sex-interactions when examining the composition and function of human microbiota.

Our growing knowledge of the host-microbiota interaction is rapidly informing translational research and therapeutic approaches to an array of chronic health conditions. Flagged as ‘the microgenderome’, gender differences and the critical role of sex hormones has been emphasized within the brain-gut-enteric-microbial axis^1^. Using an animal model, Markle *et al.* confirmed the bidirectional relationship between commensal gut microbiota, sex hormones and the immune system and provided an explanation of sexual dimorphism in Type 1 diabetes^2^. Their results revealed evidence of sex-specific microbial communities, sex-specific responses to the same microbial communities, the role of sexual maturation impacting changes to microbial communities, and evidence that microbial communities can play a protective and therapeutic role by influencing hormonal, metabolic and immune pathways. Highlighting the need to examine sex-specificity in microbial composition and function, these findings and similar^3^,^4^ suggest that intestinal dysbiosis (marked alterations in gut microbiota^5^,^6^) may play causative and consequential roles in autoimmune diseases and other health conditions^2^.

Intestinal dysbiosis and increased intestinal permeability (aberrations in the mucosal lining and musculature of the gastrointestinal tract) have been observed in the neuro-immune condition, Myalgic Encephalomyelitis/Chronic Fatigue Syndrome (ME/CFS^7^,^8^,^9^). The core feature of post-exertional fatigue and multi-systemic symptomatology reflect dysfunction of the central nervous system (CNS), immune systems and inflammatory pathways^10^,^11^. Overlapping symptom presentation and the 2:1 female-dominant incidence rates are comparative to those found in autoimmune diseases^12^. Researchers have tended to shy away from investigating this vulnerable population since the xenotropic murine leukaemia virus-related virus (XMRV) controversies^13^. However, future research is required to clarify aetiology for this complex and debilitating condition^10^. Applying the microgenderome lens to ME/CFS may provide future opportunities to elucidate unconfirmed pathophysiology and differentiate treatment pathways for this heterogeneous clinical population.

Using a cross-sectional design with a retrospective clinical data sample (*N* = 274, 68.6% female, aged 6–81 years), we were able to provide sex comparisons for a) symptom presentation; b) microbial composition and; c) interactions between microbial communities and ME/CFS symptoms (see Method for detailed explanation).

## Results

### Sex Differences in Symptom Presentation

To assess sex differences in symptom presentation, self-reported symptoms were categorised into thirteen factors; with twelve factors categorized according to the International Consensus Criteria (ICC^10^), plus a mood symptoms factor (Table S1). Patients rated symptom severity (past 7 days) and frequency (past 12 months) using a 5-point Likert scale (0–4). Impact scores (frequency × severity of symptoms) were calculated as a measure of each factor with higher scores reflecting greater impairment. Mann-Whitney tests showed sex differences for nine of the thirteen factors with measures of central tendency indicating that females were more likely to report greater impairment (Table 1). Notwithstanding possible gender differences in self-reporting or coping styles, the upregulated serotonergic response observed in female patients with CFS^14^ and evidence in parallel clinical populations, e.g., pain (osteoarthritic^15^, migraine^16^, and deep tissue^17^, irritable bowel syndrome (IBS^18^); and depression^19^ indicate that an interaction between sex steroids, neuroendocrine and immune systems is a plausible explanation for increased symptom severity and associated functional impairment in women. These results prompted investigation of pathophysiological differences.

### Sex Similarity in Microbial Composition

Comparison between sexes for each genus relied on culture-based methods of assessing faecal microbial content. Metagenomic advances provide superior detection of microbial diversity, however, culture-based methods continue to have utility to examine viable count within clinical and applied research settings^6^. Genera were quantified by viable count (frequency as per cfu/g exponent) and relative abundance (RA; ratio of genera count divided by total detectable bacteria count expressed as a percentage). Anaerobic (*Bacteroides, Bifidobacterium, Clostridium, Eubacterium,* and *Lactobacillus*) and aerobic (*Escherichia*, *Streptococcus*, *Enterococcus*) genera were investigated.

Mann-Whitney tests revealed no significant sex differences in the frequency (count) or proportion (RA) of each genus (Table S2). Additionally, sex comparisons of the total detectable bacteria count (Total Bacteria: *Mdn*_males_ = 10^10^ cfu/g, *Mdn*_*fe*males_ = 10^10^ cfu/g, *U* = 7097.5, *P* = 0.093, *r* = −0.10) and the ratio between all detectable aerobic and anaerobic bacteria (Aerobic:Anaerobic Ratio: *Mdn*_males_ = 1.21, *Mdn*_*fe*males_ = 1.10, *U* = 6844.5, *P* = 0.088, *r* = 0.10) did not differ significantly between the sexes. These results suggest sex-consistency in microbial composition within this clinical sample.

### Interactions between Microbial Community and Symptom Expression

**Figure 1:**
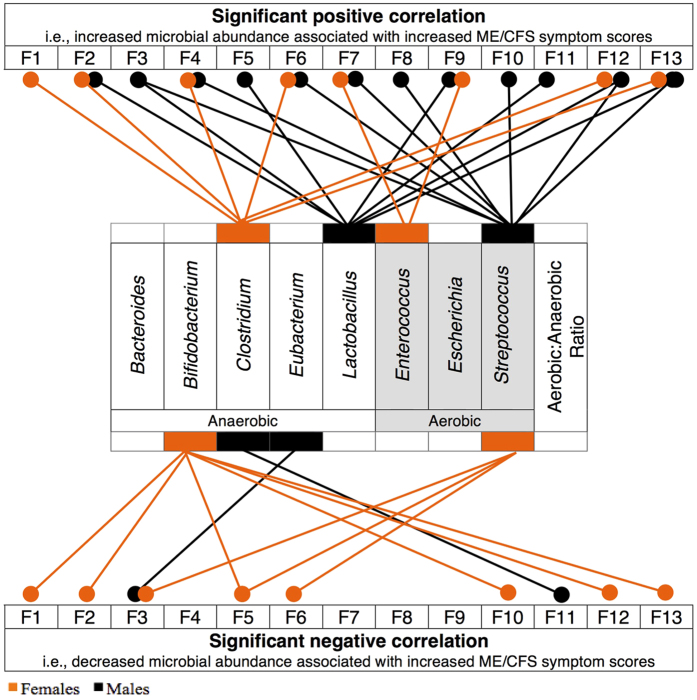
Associations between microbiota relative abundance and ME/CFS symptoms (F1–F13) for females (*n*_*range*_ = 120–170, orange), and males (*n*_*range*_ = 57–77, black). Only significant results from Spearman’s rank correlations are presented (*P* < 0.05). Anaerobic (white) and aerobic (grey) bacteria genera are distinguished. The *Aerobic:Anaerobic Ratio*: total detectable aerobic bacteria divided by total detectable anaerobic bacteria multiplied by 1000 (including but not limited to the selected genera presented above).

Spearman’s rank order correlations (*r*_*s*_) were used to investigate sex-interactions between microbial RA and ME/CFS symptom factors (Table S3). Multiple significant associations between genera and ME/CFS symptoms indicated a pattern of results diverging between the sexes (Fig. 1). The sex-specific interactions observed for *Clostridium*, *Lactobacillus* and *Streptococcus* are discussed.

### Clostridium

**Figure 2:**
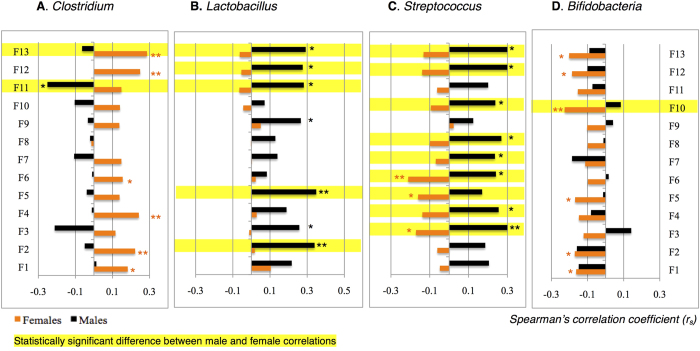
Microbial-dependent sex differences in ME/CFS symptoms (F1–F13) for females (*n*_*range*_ = 120–170, orange), and males (*n*_*range*_ = 57–77, black). Spearman’s correlation coefficient are presented showing the size of the relationship between symptom factors and the relative abundance (RA) of A. *Clostridium*, B. *Lactobacillus*, C. *Streptococcus*, and D. *Bifidobacterium*. Positive correlations indicate that an increase in microbial relative abundance was monotonically associated with an increase in symptom scores. The direction of a positive association could also be explained in reverse. Negative correlations indicate an inverse monotonic relationship between the two variables. Correlations were classified as small (0.01), moderate (0.03) and large (0.05) effect sizes^51^. *P < 0.05, **P < 0.01. *z*_*obs*_ values were calculated^52^ to examine whether there was a significant difference between male and female correlation coefficients. Statistically significant differences are highlighted when *z*_*obs*_ < −1.96 or *z*_*obs*_ > 1.96.

In females, the *Clostridium* genus was positively associated with eight of the thirteen ME/CFS symptoms. Significant small-medium positive correlations were shown for fatigue (F1: *r*_*s*_ = 0.18, *n* = 166*, p* = 0.019), neurocognitive symptoms (F2: *r*_*s*_ = 0.22, *n* = 158*, p* = 0.005), sleep (F4: *r*_*s*_ = 0.24, *n* = 164*, p* = 0.002), immunity impairments (F6: *r*_*s*_ = 0.16, *n* = 162*, p* = 0.049), total ICC symptoms (F12: *r*_*s*_ = 0.25, *n* = 123*, p* = 0.006), and total symptoms score (F13: *r*_*s*_ = 0.29, *n* = 117*, p* = 0.002). For males, an opposite association was found, with a significant negative correlation between *Clostridium* RA and mood symptoms (F11: *r*_*s*_ = −0.25, *n* = 68*, p* = 0.039). Whilst not reaching significance, a similar pattern of results was observed for pain, gastro-intestinal, and energy production/transportation impairment factors for males (Fig. 2A and Table S3).

### Lactobacillus

Figure 2B highlights the positive associations between the distribution of *Lactobacillus* and total ME/CFS symptom factors for males (F12: *r*_*s*_ = 0.28, *n* = 58*, p* = 0.036; F13: *r*_*s*_ = 0.29, *n* = 57*, p* = 0.028) in this sample (Table S3). However for females, no significant relationships were revealed between these variables. Notably, in males only, analyses recorded moderate effect sizes for neurocognitive (F2: *r*_*s*_ = 0.34, *n* = 72*, p* = 0.003) and neurosensory factors (F5: *r*_*s*_ = 0.35, *n* = 74*, p* = 0.002). Other symptoms associated with neurological impairment, including pain (F3: *r*_*s*_ = 0.26, *n* = 70*, p* = 0.031) and mood factors (F11: *r*_*s*_ = 0.28, *n* = 69*, p* = 0.019) also showed consistently significant associations and similar effect sizes for males. When considering the compositional similarity in the frequency and distribution of *Lactobacillus* across the sexes in this sample, the symptom expression differences in males may be best explained by a sex-specific response to the same microbial community.

### Streptococcus

The sex-divergent pattern of associations between *Streptococcus* levels and ME/CFS symptoms was consistent across twelve of the thirteen symptom factors (Fig. 2C and Table S3). Correlations for *Streptococcus* RA suggested opposing protective or pathogenic qualities between the sexes. For males, analyses revealed small to moderate significant positive associations between *Streptococcus* RA and pain (F3: *r*_*s*_ = 0.39, *n* = 70*, p* = 0.001), sleep (F4: *r*_*s*_ = 0.26, *n* = 74*, p* = 0.028), immunity (F6: *r*_*s*_ = 0.24, *n* = 74*, p* = 0.038), gastrointestinal (F7: *r*_*s*_ = 0.24, *n* = 73*, p* = 0.44, genitourinary (F8: *r*_*s*_ = 0.27, *n* = 77*, p* = 0.018), energy production/transportation impairments (F10: *r*_*s*_ = 0.24, *n* = 72*, p* = 0.045), ICC symptom (F12: *r*_*s*_ = 0.33, *n* = 58*, p* = 0.013), and Total symptom (F13: *r*_*s*_ = 0.31, *n* = 57*, p* = 0.017) factors. Conversely for females, there were significant negative correlations between *Streptococcus* RA and pain (F3: *r*_*s*_ = −0.17, *n* = 154*, p* = 0.034), neurosensory (F5: *r*_*s*_ = −0.16, *n* = 165*, p* = 0.040), and immunity impairments (F6: *r*_*s*_ = −0.21, *n* = 163*, p* = 0.007).

### Bifidobacterium: Possible sex consistency

Although only reaching significance in the female subgroup, analyses of *Bifidobacterium* RA provided an example of sex consistency in this sample (Fig. 2D and Table S3) and provided support for possible protective properties of these species. Significant, small negative correlations were shown between *Bifidobacterium* RA fatigue (F1: *r*_*s*_ = −0.16, *n* = 166*, p* = 0.036), neurocognitive (F2: *r*_*s*_ = −0.17, *n* = 158*, p* = 0.032), neurosensory (F5: *r*_*s*_ = −0.17, *n* = 164*, p* = 0.030), energy/production and transportation impairments (F10: *r*_*s*_ = −0.23, *n* = 164*, p* = 0.003), ICC symptoms (F12: *r*_*s*_ = −0.19, *n* = 123*, p* = 0.044), and Total symptoms (F13: *r*_*s*_ = −0.20, *n* = 117*, p* = 0.029) factors.

## Discussion

Observations in this ME/CFS sample showed a) sex differences in symptom presentation; b) sex consistency in microbial communities and; c) sex-specific interactions with gut microbiota and symptom expression. Associations between symptom level and bacterial level, in the context of sex consistency in microbial communities, imply sex-specific interactions with gut microbiota. Precise mechanisms of sex interactions can only be hypothesized because the hormonal status of patients was not available for this sample. It has been suggested that changes in microbial composition and the associated imbalance in production of estrogen receptor agonists/antagonists may contribute to immune disturbances and other symptoms observed in ME/CFS^20^. Specific bacterial taxa (*Firmicutes*, *Actinobacteria* and *Proteobacteria*) metabolise and consequently modulate homeostasis of sex steroid hormones through genes that encode hydroxysteroid dehydrogenase (HSD) enzymes^21^. Particular species within the genera *Clostridium, Bacteroides* and *Eubacterium* are known to produce the enzymes 7α– and 7β–hydroxysteroid dehydrogenase^22^,^23^, deconjugating primary bile acids enabling humans and animals to absorb cholesterol, the precursor of steroid hormones. The results from this study however question our current understanding of these processes and suggest the need to examine the host relationship with intestinal organisms at the species level of each of the three genera.

The relationship between microbiota and hormones appears bidirectional. In populations with intestinal dysbiosis, the consequence of changes to hormonal metabolism and dysregulation may help explain symptom expression and variability. In reverse, hormonal imbalances may also perpetuate intestinal dysbiosis. The Firmicutes phylum of bacteria include *Clostridium*, *Lactobacillus*, and *Streptococcus* species, all of which showed interesting sex-interactions in our sample. Prospective studies should consider obtaining hormonal status and biomarkers to examine possible interactions with microbial composition in an attempt to delineate the physiology underlying these sex-differences.

Perhaps the associations between *Clostridium* composition and some ME/CFS symptoms in females may reflect the influence of diet and variation at the species level. An increase in Firmicutes has also been associated with a more typically ‘Western diet’ with opportunistic species *Clostridium difficile* and *Clostridium perfringens* flourishing with increased refined sugar intake^24^. The sex-specific associations in the current sample raise further questions about intestinal dysbiosis in ME/CFS, particularly investigating whether higher levels of Clostridium species exacerbate neurological symptoms in females and the potential benefits of targeting treatment to restore intestinal balance.

Observations across *Lactobacillus* and *Streptococcus* genera suggest support for D-lactate as a contributing factor to symptom expression, particularly in males. This hypothesis explains the neurological symptoms of ME/CFS as a consequence of neurotoxic effects of bacterial metabolites (i.e., D-lactic acid produced by most species of *Lactobacillus* and *Streptococcus*) on the brain and nervous system^25^. Increased D-lactic acid levels have been found in the serum of CFS patients with intestinal bacterial overgrowth^7^, associated with cognitive and neurological impairments^26^, and reduced in response to treatment in a sample of CFS patients^27^. The mechanisms of a sex-specific response to D-lactic acid have not been considered.

Potential sex differences in symptom expression as a consequential or contributing factor in microbial composition have clinical and research implications. Treatment aimed at restoring intestinal homeostasis, including faecal transplants, antibiotic and probiotic therapy require consideration of individual variation and potential sex difference affecting treatment responsiveness. Clinical trials need to be designed with appropriately sized samples to enable sex comparisons. Compositional similarity within a clinical population may be falsely interpreted without considering sex interactions. Notably, the findings for *Lactobacillus spp.* in males caution against premature probiotic supplementation with D-lactate producing bacteria. However, results support the health-promoting effects of *Bifidobacteria* as observed across diverse disease states including IBS^28^,^29^, cancer^30^, anxiety and depression^31^,^32^.

In combination, our results suggest support for the microgenderome in a human clinical population. The sex-interactions observed using genera analyses do not provide specificity and prompt the need for further examination at the species level. These results call for mechanistic research to examine the role of the sex steroid interaction with microbiota in the modulation of fatigue, pain, neural and immune responses seen within ME/CFS. This is a clinically complex but potentially advantageous research population with overlapping symptomatology relevant for diverse clinical groups. Research efforts that generate phenotypes and mechanistic understanding of the human microbiome require examination of potential sex and functional differences within compositionally similar communities.

## Methods

### Setting and Participants

The methods for this study were conducted in accordance with the approved guidelines for human experimental research. Ethics approval was obtained from Victoria University Human Research Ethics Committee in May 2013 (HRE13-109). As a retrospective sample, there was no direct contact with participants. Patients obtaining faecal assessment through Bioscreen (Aust.) signed informed consent to allow their microbial results and accompanying self-reported symptoms to be used for research purposes.

The dataset included 274 patients who had signed consent to participate in research during faecal microbial assessment (FMA) through the NATA (National Association of Testing Authorities) accredited laboratory, Bioscreen. All patients received a diagnosis of CFS in accordance with the Canadian Definition Criteria^33^ or Fukuda criteria^34^ during treatment from one of the co-authors (DPL) between January 2011 and April 2013. Only the earliest available data were included when multiple FMA results were available for the same patient.

Sex representation within this study was equivalent to prevalence ratios amongst clinical ME/CFS populations^10^ with 86 male (31.4%) and 188 female (68.6%) participants. The age range of 6 to 81 years (*M* = 39.25, *SD* = 14.81) is consistently representative of the occurrence of ME/CFS across developmental stages^10^. Age was not provided for two participants. Additional demographic information or information about comorbid diagnoses were not available. Therefore, no additional exclusion criteria were applied.

### Data sources/measurement

*Faecal Microbial Analysis*. Sample collection: Prior to faecal sample collection, patients were instructed to cease antibiotic and/or probiotic treatment for four and two weeks, respectively. Patients collected a sample of their first morning bowel motion in a faecal container (anaerobic pouch system) with a perforated lid to aid anaerobiasis (achieved by activating Anaero Gen Compact (Oxoid, Thermo Fisher Scientific, Australia)). Samples were immediately transported to the laboratory in cold conditions (<12 °C) for analysis within 48 hours after collection. Laboratory protocol rejects samples subjected to inaccurate collection, transportation, anaerobiasis or refrigeration procedures. Internal quality assurance investigations validated the anaerobic transport and culture methods (see^35^).

Microbial identification and quantification: After removal from the anaerobic pouch system, all faecal samples were processed within 10–15 minutes. Between 0.5–1.0 g was transferred to 10 mL of 1% glucose-saline buffer^36^. Dilution factor was determined by the difference in the weight of the glucose-saline buffer with and without the sample. One hundred and one thousand fold dilutions (beginning from 10^−1^ to 10^−7^) of homogenised faecal samples were prepared^37^. Dilutions (10 and/or 1 μL amounts) were transferred onto dried Columbia horse blood agar (Oxoid), chromogenic medium (Oxoid), colistin and nalidixic acid blood selective agar (Oxoid), and chloramphenicol-gentamicin selective Sabouraud agar for aerobic incubation. Anaerobic incubation (4 day duration) in anaerobic jars (Oxoid) utilised pre-reduced Columbia horse blood haemin agar and Raka Ray medium. Aerobic media were incubated at 35 °C for 48 hours. A stereomicroscope was used to examine both aerobic and anaerobic culture plates for a minimum of 20 min/plate before bacterial identification. Each colony from each medium was microscopically examined and the colony/viable count were quantified for each plate. To assess purity prior to identification, similar morphotypes were sub-cultured onto horse blood agar.

Identification using MALDI-TOF MS analysis: Following overnight purity checks, index bacterial colonies were transferred to a target polished steel plate (MSP 96, Bruker Daltonics Inc.) for drying under exhaust ventilation in a Class II Biohazard Hood (Gelman Sciences Australia) at room temperature. Air-dried samples were subjected to protein extraction with 1 μL 70% formic acid (Sigma). After repeat air-drying under exhaust ventilation, samples were overlaid with 1 μL of matrix solution (saturated solution of α-cyano-4-hydroxycinnamic acid (HCCA) in a mixture of 47.5% ultra-pure water, 2.5% trifluoroacetic acid, and 50% acetonitrile). Dried samples were analysed using Microflex MALDI-TOF mass spectrometer (Bruker Daltonik GmbH, Leipzig, Germany) equipped with a 60 Hz nitrogen laser. Spectra were recorded in the positive linear mode for the mass range of 2,000–20,000 Da at maximum laser frequency. The MALDI Biotyper 3.0 software package (default settings; Daltonik GmbH, Bremen, Germany) was used to automatically analyse and measure raw spectra without user intervention. This technology can detect approximately 5000 species. The most prevalent microorganisms are quantified (viable count detection limits include anaerobes >10^8^ CFU/g, facultative anaerobes >10^5^).

**Data Used for Statistical Analysis**. Genera investigated: Anaerobic (*Bacteroides, Bifidobacterium, Clostridium, Eubacterium,* and *Lactobacillus*) and aerobic genera (*Escherichia*, *Streptococcus*, *Enterococcus*) were investigated. Species identified during FMA were classified according to genera (data provided is the combined total of species identified within each genus). Species-level analyses were not included due to the heterogeneity of species identified during MALDI-TOF MS assessment and insufficient power to correlate less common species. Whilst genera-level investigations lack specificity, some evidence suggests similar metabolic and functional capacity within taxa and genera^38^.

Justifications for selected genera: A priori selection of genera was grounded in the literature. Some of the most abundant strains of enteric microbiota within healthy human samples fall within *Bacteroides*, *Clostridium*, *Eubacterium*, and *Prevotella* as the anaerobic genera and *Escherichia* and *Streptococcus* as the aerobic genera^39^. Within infants, some of the dominant enteric microbiota include *Lactobacillus*, *Bifidobacterium* and *Streptococcus* species^40^. Whilst the abundance of specific microbiota does not necessarily equate to their purpose, function or importance^39^, they provide an initial direction for examining specific genera.

The D-lactate hypothesis and relationship between increased D-lactate levels and neurocognitive impairment^26^ further guided selection of genera investigated in this research. An association between D-lactic acidosis and an overgrowth of enteric lactic acid bacteria (including some species of *Lactobacillus, Bifidobacterium, Enterococcus* and *Streptococcus*) has been shown^7^. An Australian sample of patients with ME/CFS showed significantly higher levels of *Enterococcus* and *Streptococcus* genera viable count compared with healthy controls^7^. This study also showed variable levels of *Escherichia coli* amongst ME/CFS samples compared with controls, hence the *Escherichia* genus was also investigated.

A possible cause of D-lactic acidosis is from abnormal metabolism of carbohydrate by enteric microbiota^41^. Although not a primary byproduct, *Eubacterium* species can also produce lactic acid^42^. Evidence of dietary influences on microbial composition supported the rationale for including examination of *Eubacterium* (associated with dietary fibre and starch^43^); and *Clostridium* (associated with increased refined sugar inake^26^). Additionally, some strains of *Clostridium* have been associated with health^44^ and others with pathology^45^.

Some strains of *Lactobacillus* and *Bifidobacteria* are frequently associated with optimal health and used for probiotic supplementation^28^,^29^,^30^,^31^,^32^,^46^. Health-promoting functions of these microbiota contrast the D-lactic hypothesis and provided further justification for examining these genera.

The abundance of *Prevotella* as well as evidence of an association between colonic overgrowth and neurological symptoms^47^ suggests the need to further investigate this genus. Unfortunately, *Prevotella* species were excluded from the analysis due to variable microbial identification and quantification methods during the data collection period.

Selection of the eight genera was supported by post-hoc analyses of the current dataset showing that they accounted for large proportions of detectable microbiota in the majority of stool samples. To assess the level of representation of selected genera within this ME/CFS sample, the Total RA was calculated as the combined proportion of the eight genera investigated within the total detectable bacteria (i.e., including all genera and not specifically limited to those analyzed in this study). From the 270 samples that were assessed for both aerobic and anaerobic bacteria, the eight genera represented between 5–100% of detectable microbiota (*M* = 92.60%, *SD* = 16.80%). The most common Total RA score was 100% with 90% of the sample showing a Total RA of equal to or above 72%. Sex comparisons of the Total RA indicated similarity in representation of the eight genera investigated (*Mdn*_males_ = 99.67%, *Mdn*_*fe*males_ = 99.77%, *U* = 8529.0, *P* = 0.263, *r* = 0.068).

Count: Microbial frequency of each genus was measured in colony-forming units per gram (CFU/g). Genera exponent values were used as a measure of each microbial count per patient.

Total Bacteria: Exponent values for the microbial frequency of all detectable bacteria as measured in CFU/g.

Aerobic:Anaerobic Ratio: Total detectable aerobic bacteria divided by total detectable anaerobic bacteria multiplied by 1000. This includes all genera and not specifically limited to those selected for data analysis.

Relative abundance (RA): Percentages were calculated by dividing the viable count of each genus by the total detectable bacteria count (methods akin to^39^,^48^). The expanded whole numbers for both counts were used in this calculation.

Total RA: The sum of *RA* percentages for the eight selected genera.

Patient Questionnaire: Concurrently to faecal sample collection, patients completed an 88-item Bioscreen Patient Questionnaire (BPQ). The BPQ is used for all referring patients regardless of clinical presentation. Items address diverse symptomatology similar to the Symptom Checklist-90-Revised^49^ and Beck Depression Inventory-II^50^. Patients rated symptom severity (past 7 days) and frequency (past 12 months) using a 5-point Likert scale (0–4). Frequency scores ranked from *none at all* (0) to *extreme* (4, severity) or *constant* (4, frequency). The BPQ showed high internal consistency within this ME/CFS population (Cronbach’s α = 0.974).

ME/CFS Symptom Factors: Seventy-six BPQ items were clinically classified into 13 factors reflecting ME/CFS symptoms in accordance with the ICC (F1-F10^10^) and mood symptoms (F11; Table S1). Seventeen items were omitted that were inconsistent with the ICC, could be misinterpreted as representative of two or more factors, or did not pertain to mood symptoms. Whilst psychological or mood symptoms are not specified under the ICC, high comorbidity with depression and anxiety symptoms in the ME/CFS population provided the rationale for further investigation of mood symptoms (predominantly depressive and anxiety symptoms). An impact score (severity × frequency) was calculated for each item (possible range 0–16) and relevant items were added to form corresponding factors. As measures of combined symptomatology, an ICC Symptoms Score (summation of F1-10) and Total Symptoms Score (summation of F1-F11) were calculated.

Bias: To reduce item selection bias, the factor classification was performed according to face validity as assessed by A.W., D.B. and M.B. and confirmed by consultation with clinician, D.P.L. No changes to the factor structure were made after commencing data analysis.

As a retrospective data sample, FMA results were initially performed for clinical purposes. Hence, no *a priori* hypotheses influenced data collection methods reducing the potential for investigator bias or falsification of data.

**Statistical Methods.** Descriptive statistics were performed for all ME/CFS symptom (Table 1 and Table S1) and microbial variables (Table S2) for the total sample, males and females. No outliers were found for microbial variables. The heterogeneity of symptom scores influenced the decision to include any clusters of outliers identified by SPSS on the ME/CFS Symptom Factors. Pairwise exclusion was used for missing data. All variables defied normality, therefore, nonparametric analyses were employed.

*Examining Subgroups and Interactions*. Sex comparisons on ME/CFS symptom factors: Descriptive statistics confirmed that each symptom factor (total, females and males separately) defied normality. A series of Mann-Whitney tests were used to compare the distribution of female and male symptom scores for each factor.

Sex comparison for microbial levels: Descriptive statistics confirmed that each microbial genus (count and RA) defied normality. A series of Mann-Whitney tests were used to compare the distribution of female and male microbial levels for count and percentage distribution independently. Effect sizes were calculated using equation:


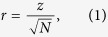


where *N* was the total sample used in the analysis. Effect sizes were classified as small (0.01), moderate (0.03) and large (0.05) according to Cohen’s (1988) guidelines^51^.

Associations between microbial level and ME/CFS symptoms: Spearman’s rank order correlations (*r*_*s*_) were used to investigate sex-interactions between microbial RA and ME/CFS symptom factors (Table S3). Missing data were excluded pairwise from the analyses. Correlations were deemed statistically significant at *P* < 0.05. Positive correlations indicated an increase in microbial relative abundance was monotonically associated with an increase in symptom scores. The direction of a positive association could also be explained in reverse. Negative correlations indicate an inverse monotonic relationship between the two variables. Correlations were classified as small (0.01), moderate (0.03) and large (0.05) effect sizes^51^.

Observed *z* scores were calculated using equation (2)^52^ to examine whether there was a statistically significant difference between the sexes for the strength of the correlation between symptom factor and microbial RA. Differences were deemed statistically significant when *z*_*obs*_ < −1.96 or *z*_*obs*_ > 1.96^52^.


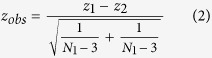


### Design Limitations and Advantages

We caution against over-interpretation of these findings considering the limitations of cross-sectional, observational research design (unable to establish causation or consequence, difficulty excluding confounding variables^53^) and categorical analysis of genera rather than species. Other genera that were not selected during this investigation may also have relevance for ME/CFS symptomatology. Technological advancement enabling 16S amplicon sequencing of viable count will be able to identify and compare a broader range of genera and species. This will then allow comparison with other ME/CFS samples (*e.g*. 20) and the ability to examine the representative nature of these results whilst considering the impact of ethnic and geographic diversity on microbial composition. Applied human research has clinical relevance^54^ and can appropriately direct the pursuits in animal investigations where mechanistic studies are needed^21^. A symbiotic, interdisciplinary approach that integrates sex differences in clinical observational data and mechanistic data will inform therapeutic directions and treatment utility.

### Additional Information

**How to cite this article**: Wallis, A. *et al.* Support for the Microgenderome: Associations in a Human Clinical Population. *Sci. Rep.* **6**, 19171; doi: 10.1038/srep19171 (2016).

## Supplementary Material

Supplementary Information

## Figures and Tables

**Table 1:** Sex differences in self-reported ME/CFS symptoms.

	ME/CFS Symptom Factors (*Possible range*)	Females			Males			*Sex Comparison*		
		*n*	*Mdn*(Range)	*M*(*SD*)	*n*	*Mdn*(Range)	*M*(*SD*)	*U*	*p*	*r*
F1.	Exertion and Fatigue (0–48)	169	31(0–48)	30.01 (*15.70*)	74	31.5 (0–48)	27.77 (*16.19*)	6806.0	0.269	0.07
F2.	Neurocognitive Symptoms (0–144)	161	47 (0–120)	50.07 (*33.52*)	72	43.5 (0–120)	44.85 (*30.13*)	6241.5	0.349	0.06
F3.	Pain Symptoms (0–208)	156	45.5 (0–179)	54.02 (*43.70*)	70	21 (0–160)	31.74 (*32.29*)	7219.0	0.000***	0.26
F4.	Sleep Symptoms (0–64)	167	29 (0–64)	30.89 (*18.66*)	74	24 (0–64)	25.51 (*18.41*)	7244.5	0.033*	0.14
F5.	Neurosensory Symptoms (0–112)	167	24 (0–103)	28.31 (*21.98*)	74	17 (0–82)	21.34 (*18.88*)	7391.5	0.015*	0.16
F6.	Immunity Impairment (0–112)	165	8 (0–72)	13.5 (*15.58*)	74	4 (0–70)	9.76 (*14.15*)	7002.0	0.068	0.12
F7.	Gastrointestinal (GI) Symptoms (0–128)	163	24 (0–113)	27.93 (*22.86*)	73	11 (0–112)	19.71 (*21.85*)	7344.0	0.004**	0.19
F8.	Genitourinary (GU) Symptoms (0–48)	170	2 (0–44)	6.54 (*9.71*)	77	4 (0–48)	8.00 (*10.91*)	5959.5	0.249	-0.07
F9.	Sensitivities (0–32)	168	12 (0–32)	12.94 (*9.71*)	72	4.5 (0–32)	7.58 (*8.24*)	8098.5	0.000***	0.27
F10.	Energy Production/Transportation Impairments (0–112)	167	22 (0–128)	30.93 (*28.67*)	72	12 (0–86)	17.78 (*19.12*)	7628.5	0.001***	0.21
F11.	Mood (0–128)	159	19 (0–113)	27.16 (*26.44*)	69	12 (0–116)	20.25c (*22.93*)	6424.5	0.040*	0.14
F12	*ICC Symptom Score* [F1-F10] (0–1008)	126	245.5 (2–826)	268.37 (*172.91*)	58	185.5 (11–607)	207.66 (*147.87*)	4480.0	0.014*	0.18
F13	*Total Symptom Score* [F1-F11] (0–1136)	120	291.5 (2–908)	264.81 (*193.41*)	57	196 (11–664)	223.72 (*161.07*)	4301.0	0.006**	0.21

Descriptive statistics, Mann-Whitney U test statistics and effect sizes (*r*) comparing symptom scores across the sexes. *P < 0.05, **P < 0.01, ***P < 0.001.
