# Supplementary material for: Support for the Microgenderome: Associations in a Human Clinical Population
Source: Sci Rep. 2016 Jan 13;6:19171. doi: 10.1038/srep19171 (PMC4725945; doi:10.1038/srep19171)
Supplement: Supplementary Information [file srep19171-s1.pdf]

Supplementary Information for

Support for the Microgenderome: Associations in a Human Clinical Population

Amy Wallis[1]*, Henry Butt[2], Michelle Ball[1], Donald P. Lewis[3], Dorothy Bruck[1]

[1]Psychology Department, Victoria University, Victoria, Australia.
[2]Bioscreen (Aust) Pty Ltd, Victoria, Australia.
[3]CFS Discovery Clinic, Donvale, Victoria, Australia.
*Correspondence to: amy.wallis@vu.edu.au

This PDF file includes:

Tables S1 to S3

	Factor Structure from BPQ Items	International Consensus Criteria *(10)*
**F1**	**Exertion and Fatigue** *(n = 3)*	A. Postexertional neuroimmune exhaustion: Compulsory
14.	Feeling low in energy or fatigued	
50.	Avoiding certain activities due to physical problems	
58.	Unusual post exertion/exercise fatigue	
		B. Neurological Impairments
**F2**	**Neurocognitive Symptoms** *(n = 9)*	1. Neurocognitive impairments
9.	Trouble remembering things	a. Difficulty processing information
38.	Having to do things slowly to ensure they are correct	b. Short-term memory loss
46.	Difficulty in making decisions	
51.	Mind going blank	
55.	Trouble concentrating	
62.	Forgetfulness	
69.	Feelings of mental tiredness or fatigue	
70.	Difficulty using words or language	
78.	Mental confusion or losing your train of thought	
**F3**	**Pain Symptoms** *(n = 13)*	2. Pain
1.	Headaches	a. Headaches
7.	Migraine headaches	b. Significant pain
10.	Frequent muscle cramps	
13.	Face pain or tenderness	
15.	Neck pain or tenderness	
16.	Shoulder pain or tenderness	
21.	Arm pain or tenderness	
22.	Leg pain or tenderness	
24.	Stiff or painful joints first thing in the morning	
25.	Joints that hurt when you move	
26.	Locking or clicking of jaw	
27.	Pain or tenderness in your lower back	
42.	Muscle soreness or stiffness	
**F4**	**Sleep Symptoms** *(n = 4)*	3. Sleep disturbances
34.	Unrefreshed or prolonged sleep	a. Disturbed sleep patterns
44.	Trouble falling asleep	b. Unrefreshed sleep
64.	Trouble waking up in the morning	
66.	Restless or disturbed sleep	
**F5**	**Neurosensory Symptoms** *(n = 7)*	4. Neurosensory, perceptual and motor disturbances
8.	Unusual muscle twitches	
29.	Tinnitus or noise in the ear	a. Neurosensory and perceptual
33.	Photophobia or dislike of strong light	b. Motor
52.	Loss of feeling, tingling or numbness of the skin	
56.	Muscle weakness or feeling of weakness in the body	
68.	Hypersensitive skin	
71.	Trouble focusing your eyes	
		C. Immune, Gastro-intestinal and Genitourinary Impairments

**F6**	**Immunity Impairment** *(n = 7)*	1. Flu-like symptoms may be recurrent or chronic and typically activate or worsen with exertion.
2.	Sinusitis or nasal congestion	
31.	Sore throat	
45.	Persistent cough	2. Susceptibility to viral infections with prolonged recovery periods.
53.	Sore or swollen lymph glands in the neck	
59.	Sore or swollen lymph glands	
65.	Sore or swollen lymph glands in the groin	
76.	Recurrent mouth ulcers	
**F7**	**Gastro-intestinal Symptoms** *(n = 8)*	3. Gastro-intestinal tract
19.	Poor appetite	
23.	Abdominal pain or tenderness	
37.	Unexplained diarrhoea	
40.	Nausea or upset stomach	
41.	Constipation	
77.	Symptoms of irritable bowel	
82.	Gastric reflux or heartburn	
83.	Cravings for certain foods	
**F8**	**Genitourinary symptoms** (n = 3)	4. Genitourinary
43.	Frequent urination	
57.	Burning or uncomfortable urination	
63.	Urgent urination	
**F9**	**Sensitivities** (n = 2)	5. Sensitivities to food, medications, odours or chemicals
17.	Allergies, intolerance or reactivity to food	
61.	Reactivity to smells or chemicals	
**F10**	**Energy Production/Transportation Impairments** *(n = 7)*	D. Energy production/transportation impairments
4.	Faintness or dizziness	1. Cardiovascular
39.	Heart pounding	
85.	Low blood pressure	
48.	Breathlessness or chest pain upon exertion	2. Respiratory
6.	Night sweats, unusual sweating while asleep	3. Loss of thermostatic stability
49.	Hot and cold spells or recurrent feverishness	4. Intolerance of extremes of temperature
75.	Cold hands or feet	
**F11**	**Mood** *(n = 8)*	*No reference made to psychological or mood symptoms on the ICC.*
3.	Repeated unpleasant thoughts	
5.	Loss of libido or sexual interest	
20.	Crying easily over your problems	
30.	Feeling blue as a results of your problem	
32.	Feeling no interest in things	
54.	Feelings of hopelessness about the future	
72.	Spells of panic related to your problems	

87.	Feeling anxious	
**F12**	**ICC Symptom Score** *(n = 63)*	
	All items from F1-F10	
**F13**	**Total Symptom Score** *(n = 71)*	
	All factor items, ICC and mood items combined.	
	**Items Omitted** *(n = 17)*	**N/A**
11.	Grinding or clenching your teeth	*Unsure about placement*
12.	Chest or heart pain	*Unsure about placement*
18.	Arthritis	*Non-ME/CFS Symptom*
28.	Feeling that your problems are disrupting your life	*Secondary*
35.	Stress from financial problems	*Secondary*
36.	Feeling that others are unsympathetic to your problems	*Secondary*
47.	Ovulation or menstruation pain	*Extensive missing data*
60.	Orchialgia or testicular pain	*Extensive missing data*
67.	Vaginal irritation or discomfort	*Extensive missing data*
73.	Sciatica or numbness/tingling down the back of the leg	*Unsure about placement*
74.	Frequently getting into arguments	*Secondary*
79.	Stressful events in your life related to your problems	*Secondary*
80.	Dermatitis	*Unsure about placement*
81.	Stress over family problems	*Secondary*
84.	High blood pressure	*Unsure about placement*
86.	Stress from work problems	*Secondary*
88.	Feelings of guilt	*Unsure about placement*

Table S1. ME/CFS symptom factor structure. Classification of BPQ items according to International Consensus Criteria *(10)*. BPQ: Bioscreen Patient Questionnaire.

			Total *N*			Males			Females			Sex Comparison		
			N	*M* (*SD*)	*Mdn* (*Range*)	*N*	*M* (*SD*)	*Mdn* (*Range*)	*N*	*M* (*SD*)	*Mdn* (*Range*)	*U*	*p*	*r*
Selected Anaerobic Genera	*Bacteroides*	Count (CFU/g)	270	9.02 (2.22)	10 (0-11)	85	8.76 (2.71)	10 (0-11)	185	9.14 (1.95)	10 (0-11)	8033.0	0.752	0.02
		RA (%)	270	58.35 (31.59)	61.45 (0-100)	85	58.21 (31.47)	58.97 (0-100)	185	58.41 (31.73)	62.30 (0-100)	7965.0	0.863	0.01
	Bifidobacterium	Count (CFU/g)	271	5.54 (3.97)	8 (0-10)	86	5.48 (4.10)	8.00 (0-10)	185	5.61 (3.91)	7 (0-10)	7910.0	0.939	-0.01
		RA(%)	271	11.44 (19.66)	0.48 (0-89.26)	86	11.81 (20.17)	0.76 (0-89.26)	185	11.27 (19.46)	0.38 (0-81.35)	7861.0	0.874	-0.001
	Clostridium	Count (CFU/g)	270	3.49 (4.15)	0 (0-10)	85	3.11 (4.07)	0 (0-10)	185	3.66 (4.18)	0 (0-10)	8416.5	0.297	0.06
		RA (%)	270	3.47 (8.26)	0 (0-53.17)	85	2.83 (6.46)	0 (0-35.45)	185	3.76 (8.97)	0.00 (0-53.17)	8380.5	0.333	0.06
	Eubacterium	Count (CFU/g)	270	5.11 (4.57)	8 (0-11)	85	5.00 (4.66)	8 (0-10)	185	5.16 (4.55)	8 (0-11)	7791.5	0.899	0.01
		RA (%)	270	17.10 (24.25)	5.30 (0-100)	85	15.51 (20.43)	4.16 (0-81.55)	185	17.84 (25.84)	5.98 (0-100)	8048.5	0.744	0.02
	Lactobacillus	Count (CFU/g)	271	3.68 (3.45)	5 (0-10)	86	3.83 (3.45)	5 (0-9)	185	3.62 (3.45)	5 (0-10)	7734.5	0.700	-0.02
		RA (%)	271	1.62 (6.76)	0.001 (0-69.75)	86	1.89 (9.25)	0.001 (0-69.75)	185	1.49 (5.24)	0.001 (0-39.20)	7766.0	0.742	-0.02
Selected Aerobic Genera	*Enterococcus*	Count (CFU/g)	274	1.81 (2.83)	0 (0-8)	86	1.78 (2.80)	0 (0-8)	188	1.83 (2.84)	0 (0-8)	8182.0	0.843	0.01
		RA (%)	274	0.44 (6.05)	0 (0-100)	86	0.13 (0.75)	0.00 (0-5.45)	188	0.58 (7.29)	0.00. (0-100)	8155.0	0.886	0.01
	Escherichia	Count (CFU/g)	247	5.84 (2.08)	6 (0-9)	86	6.10 (2.01)	6 (0-9)	188	5.72 (2.11)	6 (0-8)	7097.5	0.093	-0.10
		RA (%)	272	1.15 (7.67)	0.04 (0-87.59)	86	2.76 (13.30)	0.05 (0-87.59)	186	0.40 (1.75)	0.04 (0-22.27)	7108.0	0.140	-0.089
	Streptococcus	Count (CFU/g)	274	4.51 (2.67)	6.00 (0-9)	86	4.38 (2.80)	5.5 (0-8)	188	4.56 (2.61)	6 (0-9)	8291.5	0.726	0.02
		RA (%)	272	0.18 (0.99)	0.01 (0-11.17)	86	2.48 (1.25)	0.004 (0-11.17)	186	0.15 (0.85)	0.01 (0-10.49)	8333.0	0.576	0.03
Total Bacteria		Count (CFU/g)	271	9.73 (0.66)	10 (6-11)	86	9.76 (0.65)	10 (7-11)	185	9.72 (0.67)	10 (6-11)	7097.5	0.093	-0.10
Aerobic:Anaerobic Ratio			270	9.75 (45.94)	1.21 (0-666.73)	85	15.95 (73.40)	1.32 (0-666.73)	185	6.90 (24.50)	1.10 (0-286.85)	6844.5	0.088	0.10

Table S2. Microbial genera descriptive statistics and sex comparison results. Descriptive statistics for each microbial genus (count and relative abundance separately) across total participants, males and females. Count (CFU/g): Exponent value presented (i.e., 9.02 = $10^{9.02}$). Relative abundance (RA): ratio of genera viable count divided by total bacteria count expressed as a percentage. Total Bacteria count: exponent value of total bacteria detectable on MALDI-TOF MS assessment. Aerobic:Anaerobic Ratio: total detectable aerobic bacteria divided by total detectable anaerobic bacteria multiplied by 1000. *U*: Mann Whitney test value calculated by comparing the rank order of scores between two groups *(52)*. Effect sizes (*r*) calculated from Mann-Whitney tests comparing differences in the microbial distribution between males and females. Effect sizes were classified as small (.01), moderate (.03) and large (.05) *(51)*. No significant sex differences were shown ($P > 0.05$).

		Selected Anaerobic Genera										Selected Aerobic Genera									
		Bacteroides RA		*Bifidobacterium* RA		*Clostridium* RA		*Eubacterium* RA		*Lactobacillus* RA		*Enterococcus* RA		*Escherichia* RA		*Streptococcus* RA		Total Bacteria Count		A:AN Ratio	
		M	F	M	F	M	F	M	F	M	F	M	F	M	F	M	F	M	F	M	F
F1	$r_{(s)}$	-.02	.07	-.15	-.16*	.01	.18*	.01	.05	.22	.10	-.10	.06	.05	-.02	.21	-.05	-.07	.01	.08	.03
	P	.839	.389	.206	.036	.912	.019	.967	.511	.063	.182	.378	.434	.654	.780	.080	.534	.550	.898	.523	.700
	n	73	166	74	166	73	166	73	166	74	166	74	169	74	167	74	167	74	166	74	166
F2	$r_{(s)}$	.11	.04	-.16	-.17*	-.05	.22**	.05	.05	.34**	.02	-.08	.01	.003	-.06	.19	-.06	-.05	.07	.01	.03
	P	.343	.615	.182	.032	.676	.005	.673	.578	.003	.822	.492	.926	.981	.483	.119	.444	.682	.414	.950	.716
	n	72	158	72	158	72	158	72	158	72	158	72	161	72	159	72	159	72	158	72	158
F3	$r_{(s)}$	.000	-.06	.14	-.12	-.21	.12	-.24*	.06	.26*	-.01	-.12	.15	-.01	-.03	.39***	-.17*	-.20	.01	.14	.04
	P	1.000	.441	.241	.133	.078	.155	.044	.443	.031	.885	.326	.056	.909	.743	.001	.034	.098	.873	.255	.627
	n	70	153	70	153	70	153	70	153	70	153	70	156	70	154	70	154	70	153	70	153
F4	$r_{(s)}$	.03	.05	-.08	-.15	-.12	.24**	-.01	.01	.19	.03	-.21	-.05	.04	.04	.26*	-.14	-.08	-.03	.07	.02
	P	.809	.513	.493	.059	.329	.002	.953	.898	.107	.724	.072	.500	.707	.66	.028	.078	.515	.718	.552	.792
	n	73	164	74	164	73	164	73	164	74	164	74	167	74	165	74	165	74	164	74	164
F5	$r_{(s)}$	-.04	.001	-.01	-.17*	-.04	.14	-.08	-.004	.35**	.003	.07	.06	.05	-.02	.17	-.16*	-.08	-.02	.10	.03
	P	.752	.993	.906	.030	.746	.079	.496	.964	.002	.972	.535	.479	.664	.822	.147	.040	.498	.843	.377	.71
	n	73	164	74	164	73	164	73	164	74	164	74	167	74	165	74	165	74	164	74	164
F6	$r_{(s)}$	-.08	.000	.02	-.10	-.01	.16*	.02	.01	.08	.02	-.06	.14	.10	.004	.24*	-.21**	-.13	-.02	.07	.06
	P	.512	.999	.887	.214	.935	.049	.868	.948	.484	.772	.604	.079	.390	.964	.038	.007	.276	.827	.542	.423
	n	73	162	74	162	73	162	73	162	74	162	74	165	74	163	74	163	74	162	74	162
F7	$r_{(s)}$	.01	-.07	-.19	-.11	-.11	.15	.10	.09	.14	.003	-.14	.15*	-.08	.02	.24*	-.07	-.05	.06	-.001	.07
	P	.950	.414	.115	.156	.369	.063	.391	.280	.237	.974	.232	.049	.503	.841	.044	.383	.71	.478	.995	.359
	n	73	160	73	160	73	160	73	160	73	160	73	163	73	161	73	161	73	160	73	160
F8	$r_{(s)}$	-.14	-.02	-.01	-.10	-.02	-.01	-.02	.09	.13	-.003	-.12	.01	.02	-.03	.27*	-.10	-.22	-.03	.14	-.10
	P	.235	.848	.918	.204	.854	.861	.850	.248	.262	.971	.283	.895	.833	.670	.018	.202	.054	.709	.231	.211
	N	76	167	77	167	76	167	76	167	77	167	77	170	77	168	77	168	77	167	77	167
F9	$r_{(s)}$	-.03	.03	.04	-.10	-.03	.14	.03	.05	.27*	.050	-.18	.21**	-.06	.01	.12	.02	-.001	-.01	-.06	.10
	P	.830	.713	.734	.193	.783	.080	.791	.560	.024	.526	.138	.006	.614	.918	.301	.772	.991	.931	.614	.200
	n	71	165	72	165	71	165	71	165	72	165	72	168	72	166	72	166	72	165	72	165
F10	$r_{(s)}$	-.05	.13	.08	-.23**	-.10	.14	-.05	-.05	.07	-.04	.03	.05	.07	-.01	.24*	-.09	-.33**	-.01	.13	.04
	P	.657	.11	.482	.003	.390	.073	.706	.533	.555	.572	.776	.567	.535	.936	.045	.228	.005	.924	.264	.629
	n	71	164	72	164	71	164	71	164	72	164	72	167	72	165	72	165	72	164	72	164
F11	$r_{(s)}$	.20	.04	-.07	-.15	-.25*	.15	-.10	-.002	.28*	-.07	.03	-.03	.01	.02	.20	-.06	-.001	-.03	.01	.04
	P	.106	.66	.554	.06	.039	.065	.417	.981	.019	.421	.826	.732	.970	.828	.097	.441	.991	.761	.922	.590
	n	68	156	69	156	68	156	68	156	69	156	69	159	69	157	69	157	69	156	69	156
F12	$r_{(s)}$	-.11	.03	-.10	-.18*	-.001	.25**	.04	.02	.28*	-.05	-.05	.11	.04	-.04	.33*	-.14	-.24	.07	.08	.03
	P	.422	.742	.455	.044	.996	.006	.780	.860	.036	.552	.725	.214	.787	.651	.013	.128	.068	.424	.543	.787
	n	58	123	58	123	58	123	58	123	58	123	58	126	58	124	58	124	58	123	58	123
F13	$r_{(s)}$	-.08	.03	-.09	-.20*	-.06	.29**	.03	-.002	.29*	-.06	-.03	.11	.03	-.01	.31*	-.13	-.25	.04	.06	.06
	P	.535	.73	.507	.029	.638	.002	.839	.985	.028	.501	.815	.221	.829	.948	.017	.155	.064	.699	.644	.495
	n	57	117	57	117	57	117	57	117	57	117	57	120	57	118	57	118	57	117	57	117

Table S3. Associations between microbial composition and ME/CFS symptom factors. Spearman's rank order correlations (r_s) are shown for respective male (M) and female (F) subgroups with variable sample sizes (*n*). Relative abundance (RA): calculated from ratio of each genus viable count divided by total bacteria count expressed as a percentage. Total Bacteria count: calculated from exponent value of total bacteria detectable on MALDI-TOF MS assessment. Aerobic:Anaerobic Ratio: total detectable aerobic

bacteria divided by total detectable anaerobic bacteria multiplied by 1000. Correlations (r_s) were classified as small (.01), moderate (.03) and large (.05) *(51)*. *$P < 0.05$, **$P \leq 0.01$, ***$P \leq 0.001$.
